# Wellenreiten im Gesundheitsamt – Digitaler Wandel im Corona-Containment

**DOI:** 10.1365/s40702-021-00735-x

**Published:** 2021-05-25

**Authors:** Roland Zimmermann, Ina Zimmermann, Philipp Bornschlegl, Katja Günther

**Affiliations:** 1 Fakultät Betriebswirtschaft, insb. Wirtschaftsinformatik und Statistik, Technische Hochschule Nürnberg, Nürnberg, Deutschland; 2 Gesundheitsamt der Stadt Nürnberg, Nürnberg, Deutschland

**Keywords:** Covid-19, Containment, Öffentlicher Gesundheitsdienst (ÖGD), Prozessmanagement, SORMAS, Covid-19, Containment, Public Health Service, Process management, SORMAS

## Abstract

Das „Brechen von Infektionsketten“ ist das Ziel für den Öffentlichen Gesundheitsdienst (ÖGD) in der Pandemiebekämpfung. Diese komplexe Aufgabe benötigt digitale Unterstützung in den Gesundheitsämtern. Entgegen der landläufigen Meinung ist in den meisten Fällen dafür geeignete Software schon seit Sommer 2020 verfügbar – allerdings als nicht-standardisierte, lokale Lösungen. Viel entscheidender für die Zielerreichung ist es, die richtige Arbeitsteilung und die dazu passenden Prozesse zu definieren sowie ein enges Controlling des Kontaktmanagements zu realisieren. Erst dadurch können in kurzer Zeit viele Unterstützer in den Ämtern entsprechend der Pandemiesituation zusätzlich produktiv eingesetzt werden. In dem vorliegenden Erfahrungsbericht wird anhand eines Fallbeispiels des Gesundheitsamtes der Stadt Nürnberg aufgezeigt, wie Prozessorganisation, IT und Controlling aufeinander abgestimmt werden sollten. Diese Erkenntnisse helfen auch bei der anstehenden Vernetzung der föderal strukturierten Gesundheitsämter in Deutschland über eine zentrale Lösung (SORMAS). Denn es zeigt sich, dass lediglich die politische Vorgabe zentraler Lösungen noch lange nicht zu ihrer effektiven Nutzbarkeit führt und wiederum der Zusammenhang aus Software und Organisation berücksichtigt werden muss, um nicht Schiffbruch zu erleiden.

## Abstract (en)

Breaking “chains of infection” is the goal for the public health service during its pandemic response. This complex task requires digital support in local public health departments. Contrary to popular belief, software suitable for this purpose has been available in most cases since summer 2020 — albeit as non-standardized, local solutions. It is way more important for achieving the goal to define the right division of labor and the appropriate processes, as well as to implement close controlling of contact management. Only then can personnel be additionally deployed in the public health departments on relative short notice depending on the pandemic situation. This field report uses a case study from the city of Nuremberg’s Health Department to show how process organization, IT and controlling should be coordinated. These findings will also help during the upcoming connection of the federally structured health offices in Germany via a central solution (SORMAS). This is because the mere political specification of central solutions does not lead to their effective usability: Again, the tight relationship between software and organization must be taken into account.

## Von Wellen und Containment

Dieser Erfahrungsbericht skizziert Aktivitäten und IT-Systeme, die in der Corona-Pandemie seit 2020 von Seiten des öffentlichen Gesundheitsdienstes genutzt wurden, um die Verbreitung des SARS-CoV-2-Virus einzudämmen und zu kontrollieren. Das Gesundheitsamt der Stadt Nürnberg und seine konkreten Erfahrungen dienen als Fallbeispiel.

### Phasen der Pandemie aus deutscher Sicht

**Abb. 1:**
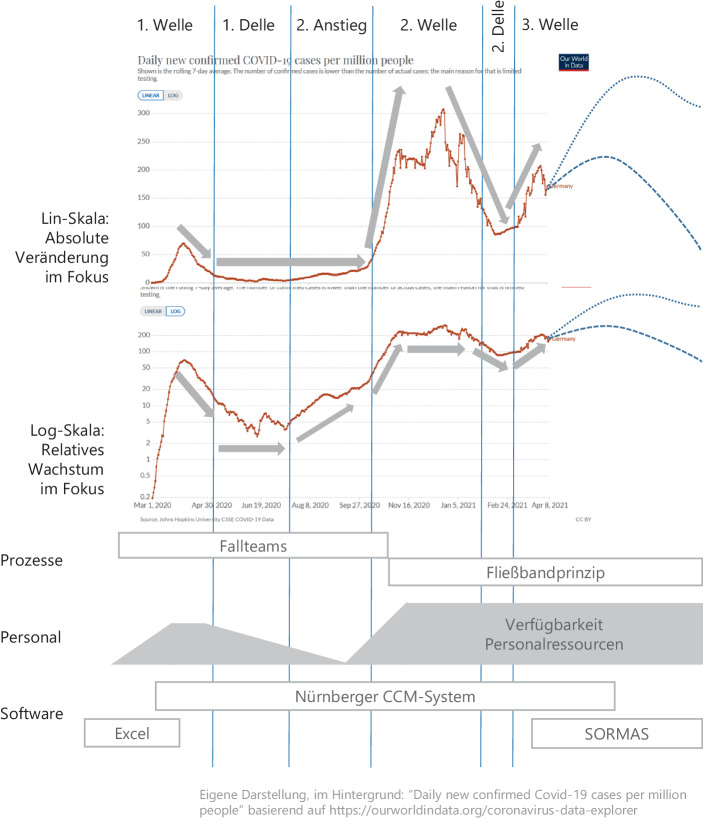
Entwicklung der Indexzahlen in Deutschland und Organisationsbereiche des Gesundheitsamtes der Stadt Nürnberg. Eigene Darstellung, im Hintergrund: “Daily new confirmed Covid-19 cases per million people” basierend auf https://ourworldindata.org/coronavirus-data-explorer

Der Haupttreiber für Maßnahmen im Rahmen der Pandemiebekämpfung in Deutschland sind die *Neuerkrankungen pro Zeiteinheit* (ergänzend normiert gemessen als 7‑Tages-Inzidenz) und die daraus resultierende Auslastung von Krankenhäusern und die Zahl verstorbener Patient*innen^1^. Abb. 1 zeigt den Verlauf der neu gemeldeten Covid19-Fälle für Deutschland seit März 2020. Die obere Grafik vermittelt auf einer linearen Skala die absolute Anzahl der Index-Fälle (IDX). Ab Mitte April 2021 ist ein Szenario-Trichter für den zukünftigen Verlauf angedeutet. Die logarithmische Skala darunter zeigt in einer verzerrungsfreien Form die relativen Änderungen der neuen Index-Fälle. Damit wird deutlich, dass sich bereits ab Juli bis September ein „versteckter“ Anstieg ergab, der ab Ende September auf einen steileren Wachstumspfad (exponentielles Wachstum) wechselte und damit zur 2. Welle führte.

### Containment-Basisprozess

Dem Containment-Prinzip liegt ein einheitlicher Prozess zugrunde, der den rund 400 lokalen Gesundheitsämtern in Deutschland vorgegeben ist: Sie haben die zentrale Aufgabe, vor Ort das Infektionsgeschehen zu beherrschen und dabei ihren Vorteil durch das Wissen über lokale Gegebenheiten im Sinne der Subsidiarität auszuspielen (Economist 2020). Alle positiven PCR-Tests auf SARS-CoV-2-Virus werden automatisch an das zuständige Gesundheitsamt gemeldet und daraufhin wird ein Prozess in Gang gesetzt, der grob dem folgenden Schema folgt (RKI 2020, 2021c):

a Indexperson (= positiv getestete Person) wird in Quarantäne gesetzt
b Befragung der Indexperson hinsichtlich ihrer Kontakte mit anderen Personen in der Zeit bis ca. zwei Tage vor Symptombeginn
c diese Kontaktpersonen werden je nach Kontaktintensität in enge Kontakte (KP‑I) oder weitere Kontakte (KP‑II) klassifiziert
d Indexpersonen und KP-I-Kontakte werden durch das Gesundheitsamt für ca. 14 Tage in Quarantäne gesetzt und täglich kontaktiert, ggf. auf Sars-CoV‑2 getestet und erst aus der Quarantäne entlassen, wenn eine Corona-Infektion ausgeschlossen werden kann.

Ziel des Prozesses ist es, die Infektionsketten zu unterbrechen und so dem Virus die Ausbreitungsgrundlage zu entziehen. Die Effektivität dieses Ansatzes ist hinlänglich bekannt, wird in asiatischen Staaten wie Singapur (Vaswani 2020) und Taiwan noch viel intensiver betrieben, wurde aber in anderen Ländern lange nicht genutzt und zuletzt erst aufgegriffen, so z. B. in den USA (New York (Associated Press 2020), Kalifornien (Becker 2020) ca. seit Mai 2020).

### Management des Containments am Fallbeispiel Nürnberg

Die verschiedenen Phasen der Pandemie sind in Abb. 1 in Relation gesetzt zu drei Aktivitätsbereichen des Nürnberger Gesundheitsamtes: dem Containmentprozess, dem Personal im Corona-Einsatz und der Softwareunterstützung für das Containment. Entsprechend strukturiert sich der Erfahrungsbericht anhand der Phasen der Pandemie, die den Lebenszyklus für das Containment-Konzept maßgeblich beeinflussen.

Im folgenden Artikel hat insbesondere das „versteckte Wachstum“ in der Phase des „2. Anstiegs“ eine große Bedeutung: Dieses Wachstum wurde im Gesundheitsamt Nürnberg durchaus wahrgenommen. Zeitgleich schrumpfte die Personalausstattung in diesem Zeitraum, so dass es zu einer Überlastsituation kam. Dies, obwohl die absolute Zahl an Index-Fällen noch auf niedrigem Niveau war (Inzidenzwerte deutlich unter 35). Dies führte in der Folge zu einer gravierenden Reorganisation (vgl. dazu die jeweiligen Abschnitte).

## Erste Welle – Spontane Digitalisierung des Containments

In der ersten Corona-Welle Ende März und Anfang April war deutschlandweit eine dezentrale, spontane, extrem agile „Digitalisierungsflut“ im öffentlichen Bereich zu beobachten, die sehr wirkungsvoll für die Beherrschung dieser Phase gewesen sein dürfte: Das Containment von Corona-Infizierten und die intensive Betreuung von Kontaktpersonen durch die Gesundheitsämter nahm rasch einen solchen Umfang an, dass eine Unterstützung für Dokumentation und Organisation durch dafür geeignete IT-Systeme nötig wurde, z. B. (Hamburg 2020), (Köln 2020), (Gernhardt 2020).

Die Kontaktnachverfolgung betrieben Gesundheitsämter bundesweit zunächst mit „Excel und (Metaplan‑)Papier“: Tatsächlich waren diese Aufgaben nicht neu für die Gesundheitsämter und im Rahmen von anderen Infektions-Ausbrüchen erprobt. Allerdings waren die Prozesse, Strukturen und IT-Systeme nicht auf die Erfordernisse einer Pandemie ausgelegt, so dass auch von den zuständigen Ministerien oftmals nur Tabellenkalkulations-Vorlagen bereitgestellt wurden, mit deren Hilfe die Kontakte von Corona-Patienten erfasst, betreut und ausgezählt werden sollten. Ein solcher Ansatz ist nicht skalierbar: Bei 10 Index-Fällen mit jeweils 10 bis 30 Kontaktpersonen ist die Ablage in separaten Excel-Dateien noch nachvollziehbar. Wenn täglich 10 oder gar 100 weitere Index-Fälle dazukommen und ein gleichzeitiger Zugriff auf die Excel-Dateien durch viele Personen nötig wird, ist ein anderes „System“ nötig.

Dem Ansturm des exponentiell sich ausbreitenden Virus ab März 2020 hielt diese „leicht-digitale Unterstützung“ daher nicht stand. In vielen Städten wurde rasch erkannt, dass nur mit skalierbaren, digitalen Lösungen die rasant wachsende Menge an Personen zu bewältigen sein würde, die in Quarantäne zu setzen, täglich zu kontaktieren und bei Bedarf auf Corona zu testen wären. In Zeiträumen, die im Normalfall nicht einmal zur Definition von Anforderungskatalogen genügen, wurden in zahlreichen deutschen Städten neue Kontaktmanagement-Systeme implementiert und ohne lange Testphasen kurzfristig erfolgreich von den Gesundheitsämtern in Betrieb genommen. Bekannte Beispiele sind: Hamburg (Hamburg 2020), Köln (Köln 2020), München (Gernhard 2020), Nürnberg (Günther et al. 2020). Ebenso wurden existierende Softwaresysteme für das Infektionsmanagement von einigen Gesundheitsämtern für das Containment genutzt, so z. B. die Softwarepakete Äskulab (Unisoft 2021) oder Octoware (EasySoft 2021). Auch vom RKI konnte eine Software genutzt werden (SurvNet@RK 2021), um Kontaktnetzwerke zu dokumentieren. Diese bot aber nur wenig Prozessunterstützung für die Ämter. Zudem gab es keinerlei zentrale Vorschläge von Bundes- oder Landesseite, die über die Nutzung von Excel-Listen hinausgingen. Tatsächlich hat das Helmholtz-Institut ebenfalls sehr früh reagiert und bereits Anfang April die ÖGD-Version von SORMAS vorgestellt (HZI 2020). Allerdings dauerte es bis November 2020 bis eine bundeseinheitliche Strategie für eine Containment-Software (SORMAS, vgl. Bundesregierung 2020) festgelegt wurde. Außerdem verteilten sich die Informationen zu verfügbaren Lösungen erst nach der ersten Welle, so dass viele Gesundheitsämter bereits ihre eigenen Lösungen nutzten und ein Wechsel ohne zentrale Vorgabe nicht wünschenswert schien.

Die wesentlichen Aufgaben eines Gesundheitsamtes zur Beherrschung des Corona-Virus müssen von einem digitalen Containment-System unterstützt werden. Anhand eines Fallbeispiels des Gesundheitsamtes der Stadt Nürnberg werden im Folgenden zentrale Funktionen und Bestandteile eines solchen Kontaktmanagement-Systems kurz skizziert.

### Nürnberger Corona Contact Management System (CCM)

Seit Februar 2020 wurden alle Daten zu Indexpersonen mit Sars-CoV‑2 an das Landesamt für Gesundheit und Lebensmittelsicherheit übermittelt, das die Daten bayernweit aggregiert und an das RKI meldet. Grundlage war eine Excel-Tabelle, die vom Bayerischen Staatsministerium für Gesundheit und Pflege bereitgestellt wurde. Damit sollten die Gesundheitsämter die Fälle und die dazugehörigen Kontaktpersonen erfassen, verwalten und die gesammelten Informationen täglich melden. Dazu wurden Teams, bestehend aus Ärzt*innen und Expert*innen gebildet. Die Dokumentation mithilfe der Microsoft-Excel-Lösung kam bereits nach wenigen Wochen Mitte März an ihre Grenzen: Die Anzahl der Mitarbeiter*innen, die die Fallbearbeitung übernahmen, wurde immer größer. Zugleich war eine parallele Bearbeitung technisch in der Dateistruktur kaum noch möglich. Außerdem benötigten immer mehr Personen einen Überblick über alle Fälle, um Aufgaben wie Abstrichmanagement (Veranlassung von Tests) und Anordnungen zur Häuslichen Absonderung (Quarantäne) zu steuern.

**Abb. 2:**
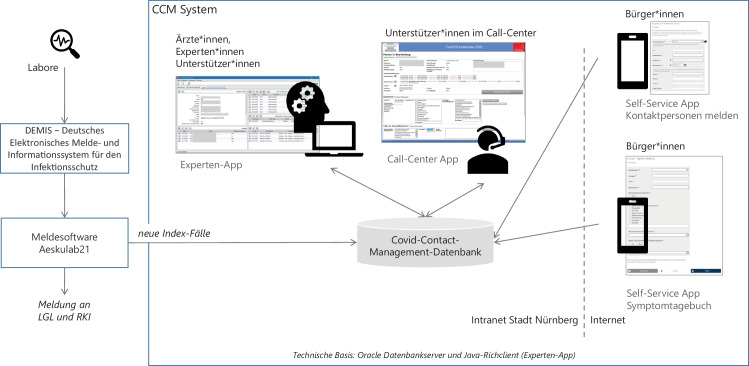
Architektur des Nürnberger Corona-Contact-Management-(CCM)-Systems

Aus diesem Grund entschloss sich das Gesundheitsamt mit dem Amt für Digitalisierung, IT und Prozessorganisation und der Technischen Hochschule Nürnberg Mitte März für die Einrichtung einer zentralisierten und skalierbaren Datenbank für die Fallbearbeitung. Es gelang innerhalb weniger Tage ein erstes integriertes Informationssystem aufzubauen, das zentrale Prozesse des Containments abbildet und das Gesundheitsamt bei der Bewältigung der Pandemieaufgaben unterstützt (vgl. Abb. 2).

Die neuen Indexfälle werden seit Januar 2021 automatisch durch DEMIS übermittelt, vorher kamen die Befunde von Laboren direkt beim Gesundheitsamt an (elektronisch + Fax). Ein Export aus dem Meldesystem Äskulab21 liefert regelmäßig neue Index-Fälle an das Nürnberger CCM-Informationssystem.

Das CCM zur Verwaltung und Bearbeitung aller Nürnberger Covid-19-Fälle und -Kontaktpersonen gliedert sich in drei Bereiche für Experten, für ein Call-Center und für Bürger*innen (Self-Service).

### Experten App

Wird ein positiver Fall an das Gesundheitsamt gemeldet, bekommt diese Person eine Identifikationsnummer und wird im Informationssystem angelegt. Die weitere Bearbeitung übernahmen in der ersten Welle die zuständigen Ärzt*innen und Expert*innen. Persönliche und medizinische Daten der Person wurden erfragt sowie jene Personen, die mit der Person in den letzten Tagen in Kontakt waren. Die Fallbearbeiter*innen sprachen mündlich die häusliche Absonderung für den positiven Fall sowie für die Kontaktpersonen aus und übertrugen die Daten in das Informationssystem.

**Abb. 3:**
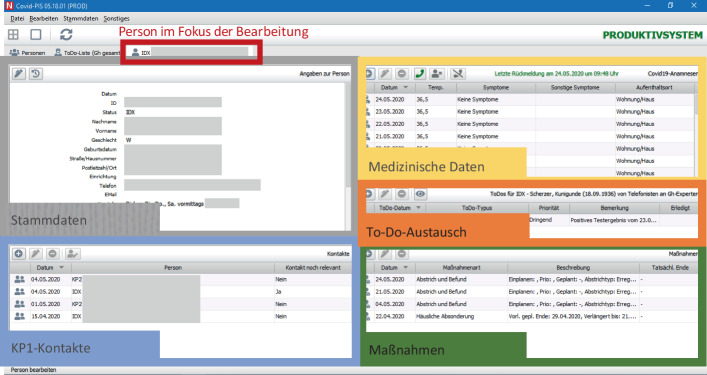
Hauptfenster der Experten-App zur Unterstützung der wesentlichen Corona-Prozesse des Gesundheitsamtes: Kontaktverfolgung, tägliche medizinische Überwachung, Maßnahmeneinleitung (Quarantäne, Tests, etc.), ToDos

Die Experten App (vgl. Abb. 3) unterstützt zahlreiche Maßnahmenarten, die in der weiteren Fallbearbeitung benötigt werden. Alle positiven Fälle sowie die Kontaktpersonen werden täglich angerufen, um Informationen zum Gesundheitszustand zu erfahren. Diese Informationen werden in einem täglichen Anamnesebogen eingetragen. Außerdem können weitere mögliche Aktionen durchgeführt werden, wie z. B. die häusliche Absonderung (Quarantäne), die Organisation eines Tests für Kontaktpersonen, eine Ausnahmegenehmigung für medizinisches Personal oder das Senden einer schriftlichen Anordnung. Alle Maßnahmen können über das Informationssystem angeordnet und überwacht werden. Hinter allen Maßnahmen stehen ggf. weitere Teams, die die Organisation zum Beispiel von Testungen oder das Versenden von Anordnungen übernehmen. In der zugrundeliegenden Datenbank werden alle Maßnahmen dokumentiert, so dass alle Beteiligten jederzeit den Status und die Historie von Erkrankten sowie Kontaktpersonen einsehen und sich damit bei Bedarf auch gegenseitig vertreten können.

### Call-Center App

Um die täglichen Anrufe zur Überwachung der positiven Fälle sowie der Kontaktpersonen zu bewältigen, wurde ein ergänzendes, in der Handhabung vereinfachtes Web-Informationssystem, programmiert. Damit konnten schon im April auch kurzfristig Personen für die Überwachung angelernt und eingesetzt werden. So haben bereits im April ca. 100 externe Unterstützer*innen für das Gesundheitsamt der Stadt Nürnberg gearbeitet. Die Aufgabenteilung sah in dieser Phase vor, dass der erste Kontakt zum positiven Fall und seinen Kontaktpersonen immer von den zuständigen Ärzt*innen und Expert*innen getätigt wurde. Der regelmäßige Kontakt bis zum Ende der Quarantäne wurde dann von Unterstützer*innen übernommen. Das Web-Informationssystem bietet einen Überblick über die wichtigsten Daten einer Person. Den Unterstützer*innen kommt die Aufgabe zu, täglich den Gesundheitszustand der jeweiligen Person zu erfragen und in das System einzutragen. Wird eine neue Maßnahme, wie beispielsweise ein Test oder auch ein Rückruf durch Ärzt*innen gewünscht, so kann diese Information in Form eines „ToDos“ über dieses Tool weitergegeben werden.

### Self-Service Apps

Betroffene Personen von SARS-CoV‑2 können auch selbstständig den Kontakt zum Gesundheitsamt halten. Sind sie im System aufgenommen, so bekommen sie eine Identifikationsnummer während der Quarantänezeit und können täglich über ein E‑Formular Daten zu ihrem Gesundheitszustand digital senden. Diesen Service nutzen rund 20 % der aktiv zu überwachenden Personen. Die Informationen werden automatisch im CCM-System verbucht. Meldet sich eine Person nicht täglich oder entwickelt eine Kontaktperson relevante Symptome, so erhalten die Fallbearbeiter*innen einen Hinweis und nehmen telefonisch Kontakt zur Person auf.

Über ein weiteres Formular können Index-Personen zu Beginn ihrer Erkrankung die Kontaktpersonen systematisch aufführen, die vom Gesundheitsamt zu kontaktieren sind. Dadurch beschleunigt sich der Prozess, bis auch die Kontaktpersonen (KP I) erreicht und in Quarantäne gesetzt werden, so dass Infektionsketten schneller unterbrochen werden.

### Controlling für das Containmentmanagement

**Abb. 4:**
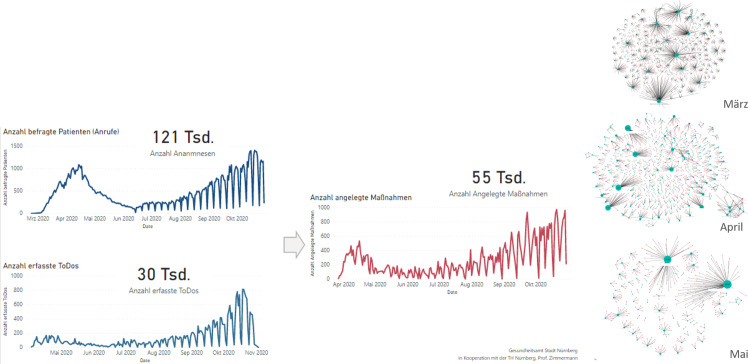
Controlling der Aktivitäten (*links*, Stand 30.10.2020), Kontaktnetzwerke im Zeitverlauf (*rechts*)

Auf Basis der CCM-Datenbank konnte das Management der Pandemie-Aktivitäten durch das Gesundheitsamt bereits seit April detailliert kontrolliert und gesteuert werden (vgl. Abb. 4). So wurden alleine bis Ende Oktober mehr als 120.000 Telefonate geführt, in denen der tägliche Gesundheitszustand von Indexpersonen und ihren Kontaktpersonen in Nürnberg erhoben wurde. Dabei mussten über 55.000 Maßnahmen wie Tests, Quarantänen und Anordnungen erfasst und mehr als 30.000 ToDos bearbeitet werden. Zugleich lassen sich auf Basis der erfassten Kontaktdaten auch die zugehörigen Kontaktnetzwerke darstellen (vgl. rechts in der Abb. 4). So ist unschwer zu erkennen, dass im März pro Indexperson noch deutlich mehr Kontakte bestanden als im April, nachdem der Lock-Down seine Wirkung entfaltet hatte.

## Erste Delle – Corona-App, Konsolidierung und Personalrückbau

### Zwei Welten: Corona-App und Containment

Seit Mitte Juni 2020 ist die Corona-Warn-App verfügbar: Dezentral und anonym verspricht sie, Kontaktketten zu berücksichtigen, die andernfalls unentdeckt blieben: im Bus, beim Einkaufen, im Restaurant oder wo sich Menschen ansonsten anonym begegnen. Und gleichzeitig will sie die Freiheit und Selbstbestimmung der Bürger*innen nicht einschränken – anders als dies bei zentralisierten Warn-Apps der Fall ist (Spiegel Netzwelt 2020 und Kluth 2020), deren Effektivität allerdings unbestritten ist. Dabei ist nicht zu übersehen, dass diese App erst nach dem Abebben der ersten Corona-Welle verfügbar war und somit eine Wirkung erst ab der zweiten Welle entfalten konnte.

Die Corona-App erreicht – genügende Verbreitung vorausgesetzt – insbesondere die Kontakte, die von den Gesundheitsämtern nicht erreicht werden können, weil sie vollkommen anonym verlaufen sind (z. B. im Bus) und ergänzt damit das Kontaktmanagement der Gesundheitsämter. Konkret bedeutet das: Sobald jemand ein erhöhtes Risiko über die App gemeldet bekommt, soll die Person sich möglichst sofort in ihren Kontakten beschränken und bei einem Arzt oder direkt dem Gesundheitsamt melden. An diesem Punkt wird die Person also erstmals und freiwillig als eine spezielle Form von „Verdachtsperson“ ggf. sichtbar, für die dann geklärt werden muss, wie stark der Verdacht tatsächlich begründet ist.

Im Verlauf des Jahres 2020 hat die App mehrere Erweiterungen erfahren, so dass sie nun auch Ergebnisse eines Labortests auf Wunsch an die Nutzer*innen übermittelt und Nutzer*innen freiwillig ein Kontakttagebuch führen können, so dass sie im Falle einer Infektion sofort auskunftsfähig sind gegenüber ihrem Gesundheitsamt (RKI 2021a). Inzwischen wurde die App fast 27 Mio. mal installiert, was jedoch nicht ausreicht für ein flächendeckendes Netzwerk in Deutschland. Gründe für die begrenzte Nutzung sind vielfältig, z. T. fehlt manchen Nutzergruppen (insbesondere älteren Risikopersonen) die technische Fähigkeit, eine App auf ihrem Smartphone zu installieren (Uni Mannheim 2020). In der gleichen Studie zeigte sich jedoch auch, dass die generelle Bereitschaft zur Installation der App Ende 2020 unter 40 % lag. Dennoch werden täglich Infektionen über die App gemeldet und Kontaktpersonen über ihr Risiko informiert (RKI 2021b).

### Auftreten zentraler Ansätze für Kontaktmanagement-Systeme

Anhand der frühen individuellen Systeme aus Hamburg, Köln, München und Nürnberg wurden bereits im Juni 2020 zentrale Anforderungen an Containment-Systeme abgeleitet und mit Forderungen an eine zentralisierte Lösung verbunden (Günther et al. 2020). Aus den öffentlich zugänglichen Unterlagen (Hamburg 2020; Köln 2020; Gernhardt 2020) wurden folgende zentrale Fachfunktionen abgeleitet:

Neben der grundlegenden Kontaktdokumentation, die alle Angaben zu erkrankten Personen und ihren Kontaktpersonen umfasst, steht das Individuum mit seiner Quarantäne, täglichen Anamnesen und möglichen Testungen im Mittelpunkt. Ebenso müssen Institutionen wie z. B. Besonderheiten in Alten- und Pflegeheimen, aber auch Gemeinschaftsunterkünften oder Organisationen der kritischen Infrastruktur abgebildet werden. Weitere Anforderungen kamen dann im Verlauf des Jahres 2020 hinzu, so z. B. der Umgang mit Reiserückkehrern und zuletzt die Integration von Mutationsvarianten des Virus oder die Dokumentation von Impfungen.

Tatsächlich werden viele dieser fachlichen Anforderungen sowie die begleitenden IT-Anforderungen nach *Standardisierung, Cloud-basiertem Hosting, Open-Source-Ansatz* und *Vernetzung der Gesundheitsämter* mit der Softwarelösung SORMAS des Helmholtz-Institutes erfüllt (HZI 2020). Leider wurde diese Lösung erst im November 2020 als verbindlich für Deutschland von den Regierenden des Bundes und der Länder bestimmt (Bundesregierung 2020).

Insofern wurde in den Sommermonaten nach der ersten Welle lokal mit den etablierten Lösungen weitergearbeitet, die Prozesse wurden optimiert und zugleich konnte in vielen Fällen Personal wieder in seine eigentlichen Aufgabenbereiche zurückbeordert werden (s. unten). Unter diesen Umständen ist es naheliegend, dass keine freiwilligen Migrationen auf ein zentrales System erfolgten, zumal gar keine Festlegung für eine zentrale Variante existierte und von daher keine Orientierung geboten war, auf welche Lösung zu setzen es sich gelohnt hätte. So hat das Land Bayern zwar eine eigene landesspezifische Lösung (*BaySIM*) Ende April angekündigt (STMD 2020), die ab Juli 2020 verfügbar war und auf die 35 Gesundheitsämter in Bayern migriert sind (Bayerischer Landtag 2020). Aber die größten langfristigen Netzwerk- und Synergieeffekte für die Pandemiebekämpfung und auch die Weiterentwicklung einer Softwarelösung bietet naturgemäß eine bundeseinheitliche Lösung. Von daher ist der Entschluss der Ministerpräsidentenkonferenz von Mitte November 2020 konsequent – nur leider sehr spät. Rückblickend ist der Sommer 2020 ein ungenutztes Zeitfenster geblieben, das für eine Vereinheitlichung der digitalen Containment-Unterstützung hätte genutzt werden können.

Im Frühjahr 2021 sieht dagegen die Pandemielage deutlich schlechter aus als noch im Sommer 2020 (vgl. Abb. 1) und es haben sich zudem sehr viel stärkere Lock-In-Effekte für bestehende Lösungen ergeben, so dass eine vollständige Migration von lokalen Containment-Lösungen nur noch mit sehr viel mehr Aufwand als noch im Sommer 2020 möglich ist. Dieser Mehraufwand steht zusätzlich im Konflikt mit der angespannteren Gesamtlage aus Inzidenzhöhen und neuen Mutationen im Frühjahr 2021.

### Personalrückbau im Corona-Containment

Ab Ende April 2020 gingen die Neuinfektionszahlen derart stark zurück, dass die Personalkapazitäten für das Containment nicht mehr in vollem Umfang benötigt wurden. Zugleich wurde Personal, dass abgeordnet worden war zur Containment-Unterstützung nun zu seinen Heimatstellen zurückgeholt; Überstunden und nicht-genommene Urlaubstage mussten reduziert werden.

**Abb. 5:**
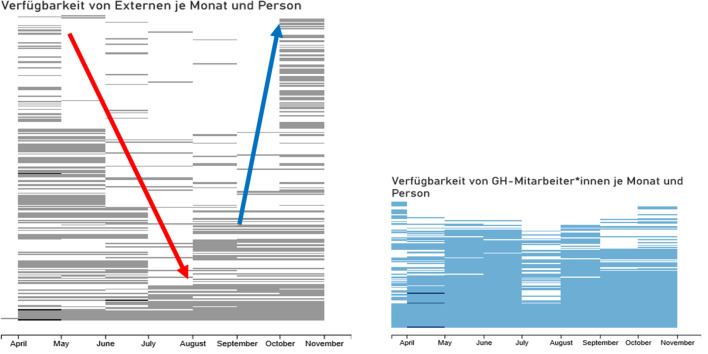
Personalentwicklung April bis Oktober 2020, Fallbeispiel Nürnberg

Abb. 5 verdeutlicht diesen Personalabbau am Fallbeispiel Nürnberg. Er erfolgte bei „externem“ Personal, also allen Personen, die nicht originär im Gesundheitsamt tätig sind. Dagegen ist ersichtlich, dass es nur wenig Reduktion von internem Personal gab (rechte Seite), obwohl zunehmend auch wieder Regelaufgaben durch das Gesundheitsamt wahrgenommen werden mussten.

Der Personalabbau setzte sich bis Juli fort und erst im August wurde wieder teilweise mehr Personal eingesetzt. Seit Oktober (letzte Spalte der linken Grafik) sind deutlich mehr Personen verfügbar. Allerdings ist deren tatsächliche Einsetzbarkeit zeitverzögert, da zunächst ein Anlernen stattfinden muss. Grund: Es handelt sich zu großen Teilen um Personen, die bislang nicht im Containment-Prozess eingesetzt wurden. Dies ist je Zeile an den leeren linksseitigen Spalten im Vergleich zu der ganz rechten Spalte ablesbar. Gründe sind Personalfluktuation und die eingeschränkte Wiederverfügbarkeit von freiwilligen Helfer*innen.

## Zweiter Anstieg – Krise der Fallteam-Struktur

### Hintergründe der Krise des bisherigen Containment-Prozesses

**Abb. 6:**
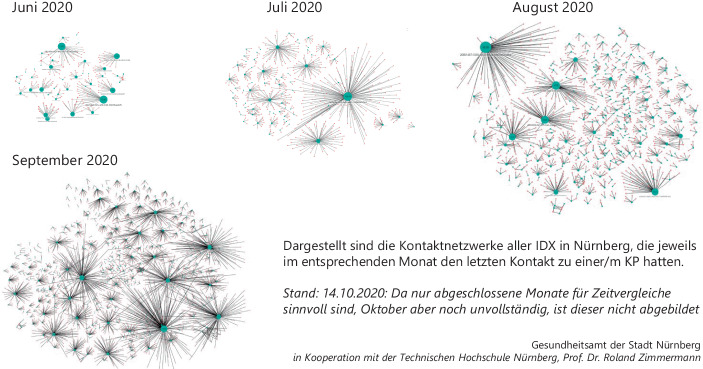
Kontaktnetzwerke in Nürnberg Juni bis September 2020

Bereits Ende September 2020 wurden im Gesundheitsamt Nürnberg Beobachtungen gemacht, die zeigen, dass die schleichende Steigerung der Indexfälle auf niedrigem Niveau in Gesundheitsämtern schon zu merklichen Problemen führen konnte:

1. Die Kontaktintensität, d. h. wie viele Kontaktpersonen ein Index hat, ist seit Juni in Nürnberg deutlich gestiegen (vgl. Abb. 6). Parallel dazu stieg die Anzahl der Indexpersonen bereits wieder sichtbar.
2. Eine Analyse der Regelungsdichte, d. h. der Anzahl zu beachtender Regelungsschreiben (z. B. Ministerielle Schreiben des bayerischen Gesundheitsministeriums (GMS) und ergänzende Formulare) zeigt, dass seit März bis Ende Oktober 2020 ca. 700 Schreiben die Arbeit der Fallteams erheblich komplexer gemacht haben (Stand Januar 2021 sogar ca. 1400 Schreiben).
3. Zeitgleich blieb die Verfügbarkeit externer Unterstützer*innen im August und September auf relativ niedrigem Niveau (vgl. nochmals Abb. 5).
4. Das verfügbare Unterstützungspersonal wechselte und angelernte Personen gingen dadurch verloren. Die Trainingslast für neues Personal lag beim Stammpersonal des Gesundheitsamtes.
5. Ab etwa der KW 43, 2020 stiegen die Fallzahlen derart schnell, dass die Fälle nicht mehr wie bisher zu bearbeiten waren.

### Organisationsform „Fallteams“ von März bis Oktober 2020

In der Zeit von März bis Oktober 2020 wurden Indexpersonen und ihre Kontaktpersonen jeweils von Beginn (d. h. dem Zeitpunkt des positiven Befundes der Indexperson) bis zur Entlassung der Kontaktpersonen aus der Quarantäne vollständig von einem für sie dauerhaft zuständigen Fallteam unter der Leitung von Ärzt*innen betreut.

**Abb. 7:**
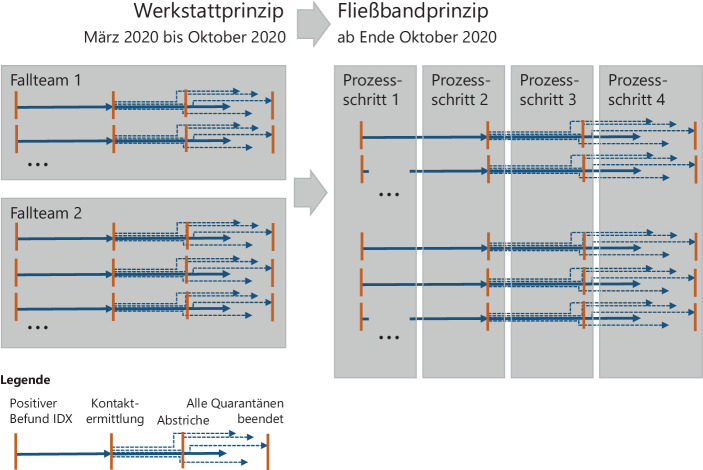
Organisationsformen des Containments im Gesundheitsamt der Stadt Nürnberg

Abb. 7 zeigt links schematisch die Organisation des Containment-Prozesses nach Fallteams. Dargestellt ist ein Index (durchgezogener Pfeil) von positivem Befund bis zum Ende der Quarantänezeit. Kontaktpersonen eines Index werden durch die Kontaktermittlung bekannt und werden, hier als gestrichelte Pfeile gekennzeichnet, ebenfalls bis zu ihrem eigenen Quarantäneende durch die Fallteams betreut. Im Verlauf der Betreuung werden in vielen Fällen Abstriche (Tests) durch das Gesundheitsamt angeordnet.

Im Rahmen der Fallteam-Organisationsform wurden zwei Umorganisationen vorgenommen:

1. Im Mai/Juni und dann wieder seit Mitte August hat eine zentrale Gruppe von Unterstützer*innen über alle Fallteams hinweg im Sinne eines „innerstädtischen Call-Centers“ die täglichen Anrufe während der Quarantäne durchgeführt.
2. Zudem wird seit Mai ein Online-Symptomtagebuch angeboten, das von Internet-affinen Personen gerne genutzt wird und damit das Call-Center entlastet.

### Verstärkende Kausalzusammenhänge lassen Fallteam-Struktur kollabieren

**Abb. 8:**
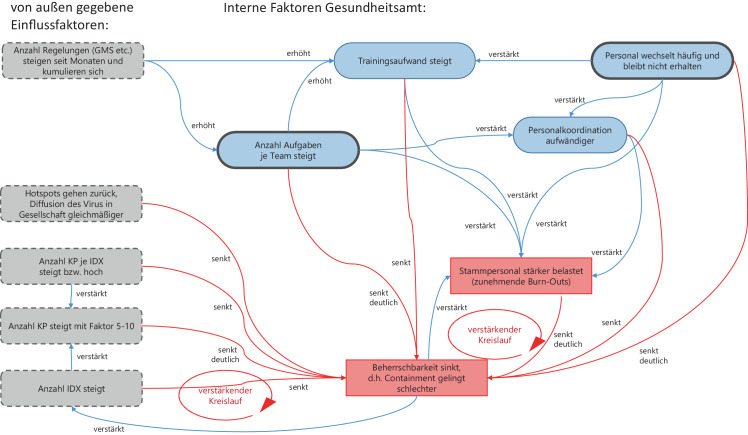
Einflussfaktoren auf die Beherrschbarkeit des Containments in der Organisationsform „Fallteams“

Allerdings führten die geänderten Rahmenbedingungen dazu, dass diese Organisationsform bei ansteigenden Indexzahlen, schwankender Personalversorgung und gleichzeitig deutlich ansteigender Regelungsdichte eine zunehmend schlechtere Beherrschbarkeit des Containments erzielte. Die Gründe sind in dem Kausaldiagramm (vgl. Abb. 8) detailliert:

1. Externe Einflussfaktoren (links gestrichelt und grau dargestellt), wie die Anzahl der ministeriellen Schreiben (GMS) aber auch die Komplexität der Fälle etc. lassen sich nicht vom Gesundheitsamt beeinflussen, haben aber einen negativen Effekt auf die Beherrschbarkeit des Containments.
2. Wechselndes Personal verstärkt die Notwendigkeit für Training sowie Personalkoordination und senkt damit die Beherrschbarkeit.
3. Zentral ist die „Anzahl Aufgaben je Team“: Sobald diese steigt, und das ist insbesondere durch die Vielzahl an GMS und damit einhergehenden Spezialisierungen in der Fallbearbeitung zu erklären, werden zahlreiche weitere Faktoren negativ beeinflusst. Dies sind:
  - Trainingsaufwand steigt
  - Personalkoordination wird komplexer
  - Stammpersonal wird in der Folge stärker belastet
  Im Ergebnis wird über die kausalen Beziehungen zwischen den Faktoren ein starker negativer Einfluss auf die Beherrschbarkeit des Containments ausgeübt, solange gilt, dass innerhalb eines Fallteams der gesamte Prozess von „Positivem Index-Befund“ bis „Quarantäne-Ende aller Kontaktpersonen“ als Kompetenzen vorhanden sein müssen.
4. Die steigende „Anzahl Aufgaben je Team“ führt in der Kombination mit häufig wechselndem externen Personal zu einem mehrfach-verstärkenden Effekt und einer Überlastung des Stammpersonals des Gesundheitsamtes.

#### Fazit

Die Beherrschbarkeit des Containments kann nur verbessert werden, wenn die Anzahl der Aufgaben je Team drastisch reduziert wird und das Personal seltener wechselt.

## Zweite Welle – Fließbandprinzip für massive Skalierbarkeit des Containments

### Von der Werkstattfertigung zum Fließband

Aus dem Kausaldiagramm ist zu schließen, dass eine Verbesserung der Beherrschbarkeit und eine Entlastung des Stammpersonals möglich sind, wenn die Aufgabenspanne je Team reduziert wird. Dazu ist folgender Vergleich aus der Betriebswirtschaftslehre angebracht:

- Die bisherige Organisationsform der Fallteams ist mit einer „Werkstattfertigung“ zu vergleichen, die den Vorteil hat, dass kundenindividuelle Wünsche von Spezialisten in der Werkstatt erfüllt werden. Diese Spezialisten haben ein sehr breites Kompetenzprofil. Sie sind rar, brauchen eine lange Anlernphase und das Gesamtsystem kann nur eine begrenzte Zahl an Kunden zufriedenstellen.
- Soll hingegen eine sehr große Anzahl möglichst gleichartiger Wünsche („Produkte“) erfüllt werden, so wird Fließbandfertigung genutzt, die durch extreme Arbeitsteiligkeit geprägt ist. Komplexe Schritte werden in kleine Einzelschritte zerlegt und damit für einzelne Personen leicht erlernbar, da sie ggf. nur einen oder zwei solcher Einzelschritte beherrschen müssen.

Voraussetzung für ein „Fließband-Prinzip“ ist ein tiefes Prozessverständnis derjenigen, die den Gesamtprozess strukturieren, um eine adäquate Zerlegung in Teilprozessschritte definieren zu können. Dieses Prozesswissen war zu Beginn der Pandemie vermutlich nirgendwo in Deutschland verfügbar, sondern die Erfahrungen wurden in allen Gesundheitsämtern, so auch in Nürnberg, erst in den Monaten seit März 2020 gemacht. Deshalb wird vermutet: Ein „Fließband-Prinzip“ war in der ersten Welle mangels Erfahrungswissen kaum realistisch umsetzbar. Zudem bestand aufgrund der geringen Fallzahlen kein Handlungsdruck in der darauffolgenden Sommerzeit, denn ein „Werkstattprinzip“ ist für geringe Mengen durch seine Flexibilität durchaus gut geeignet.

Die Notwendigkeit zum „Fließband-Prinzip“ zu wechseln, wurde erst mit dem drastischen Anstieg der Indexfälle seit Oktober 2020 greifbar, auch wenn sich durch die stärkere Spezialisierung der Fallteams die Belastungssituation für das Stammpersonal bereits über den Sommer verschlechterte. Bei vielen Indexfällen ist die „werkstattbasierte“ Fallbearbeitung auch mit mehr Personal kaum noch zu bewältigen, denn das Anlernen neuen Personals dauert zu lange.

Die Herausforderung dennoch massiv Personal aufzubauen, lässt sich wie folgt charakterisieren: Wenn die meisten der Gesundheitsämter vom Umfang des auf Infektionsbekämpfung spezialisierten Personals im Februar zumeist die Größe von kleinen Start-Ups hatten, so mussten innerhalb kürzester Zeit Strukturen entwickelt werden, die denen eines typischen mittelständischen Unternehmens mit oft mehreren hundert Mitarbeiter*innen entsprechen. Schon in monetär stark motivierenden Bereichen der Privatwirtschaft ein schwieriges Unterfangen, das nicht leichter wird, wenn das Ansinnen in den öffentlich-rechtlichen Bereich verlegt wird und viele der Mitarbeiter*innen nicht freiwillig, sondern qua Abordnung zum Dienst gesendet werden.

### Containment-Prozess 2.0

**Abb. 9:**
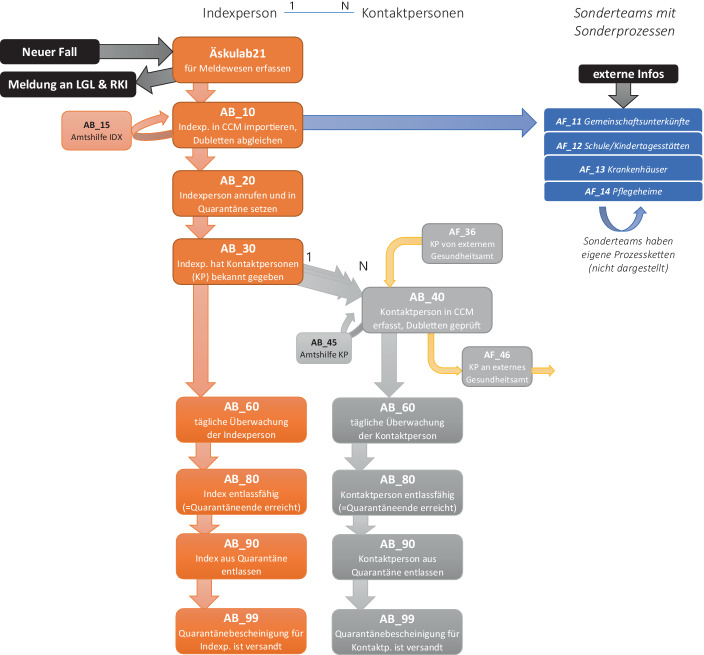
Prozess-Detailschema mit Meilensteinen und Teamstrukturen (Stand Dezember 2020)

Abb. 9 verdeutlicht den geänderten Containementprozess auf Basis sehr kleiner, klar definierter Prozess-Teilschritte.

Für jeden Teilabschnitt ist ein eigenes Team verantwortlich, es gibt klar definierte Schnittstellen durch Meilensteine, die am Ende jedes Prozess-Teilschritts vergeben werden. Die Meilensteine sind von Anfang an in 10er-Schritte untergliedert worden, um bei Bedarf auch neue Teilschritte einfügen zu können. Der Prozess von AB-10 bis AB-99 stellt den Basisprozess für Bürger*innen dar. Ergänzend sind Prozessvarianten in ähnlicher Form für Sonderteams definiert worden, so z. B. für den Heimbereich oder auch Krankenhäuser (rechts in Abb. 9). Die Bildung dieser Sonderteams, welche für die Bearbeitung von Sonderfällen zuständig sind, ist aufgrund der fachlichen Komplexität der Fallbearbeitung in bestimmten Settings sinnvoll.

Dieser Prozess wurde Ende Oktober inklusive dazugehöriger Team-Aufgabenbeschreibungen definiert, so dass ab Montag dem 26.10.2020 die Besetzung der Teams und die dann folgende Umorganisation gestartet wurde. Die Bearbeitung der Index- und Kontaktpersonen hat sich seitdem deutlich beschleunigt (vgl. Durchlaufzeitanalyse).

Ein großer Vorteil für die Umsetzung des neuen Prozesses war die Flexibilität des CCM-Systems. Die bisherigen Zuständigkeiten (Personen) wurden um die abstrakten Meilensteine ergänzt, so dass die Zuständigkeit nun nicht mehr Fallteam-spezifisch, sondern je nach abgeschlossenem Meilenstein vergeben wird. Die Meldung der Fallzahlen an das LGL erfolgt weiterhin über die Software Aeskulab21.

Innerhalb des CCM-Systems können alle Beteiligten jederzeit ihren „Arbeitsvorrat“ als Listen abfragen, denn jedes Prozess-Team bearbeitet alle die Personen, die den zuvorliegenden Teilschritt erfolgreich abgeschlossen haben. Das bedeutet, dass z. B. das Team für „AB_20“ die Liste aller in der Datenbank neu erfassten Indexpersonen aufruft, also derjenigen Personen, die den Meilenstein AB_10 erreicht haben. Sobald die Aufgaben für den Meilenstein AB_20 erledigt sind (u. a. in Quarantäne setzen der Indexperson), wird die Person auf den Meilenstein „AB_20“ gesetzt und kann vom Folgeteam (hier Team für AB_30) übernommen werden. So ist eine kontinuierliche Weitergabe der Index- und Kontaktpersonen über alle Prozessschritte hinweg gewährleistet.

Das Training für neue Mitarbeiter*innen erfolgt nur für einzelne Arbeitsschritte, so dass die Aufgabenvielfalt deutlich reduziert wurde und sehr viel leichter neue Mitarbeiter*innen in den Prozess eingebunden werden können. Jeder Prozessschritt ist detailliert dokumentiert und über ein zentrales Online-Portal (WIKI-System) für alle jederzeit verfügbar. Damit hat sich die Skalierbarkeit des Containments für Nürnberg deutlich verbessert.

### Prozessanalysen für besseres Containment

**Abb. 10:**
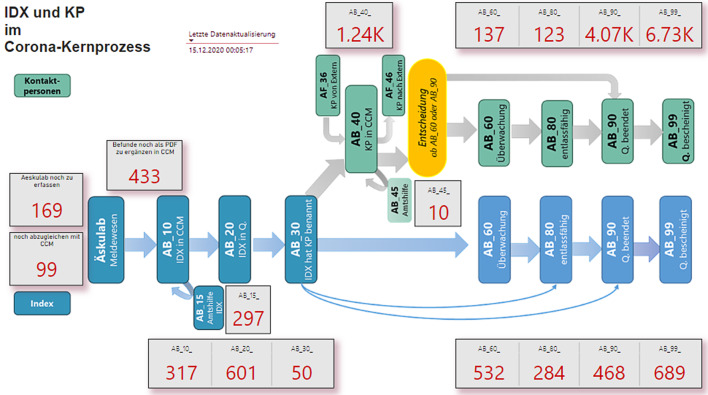
Prozessdashboard für den Corona-Kernprozess (aktuelle Arbeitsvorräte)

Die explizite Prozessdokumentation bietet vollständige Transparenz über den gesamten Containment-Prozess. Da für jede Index- und Kontaktperson in der Datenbank die Meilensteinveränderungen mitprotokolliert werden, ist der Arbeitsvorrat sowie die Leistung je Prozessschritt zu jedem Zeitpunkt für alle Prozessbeteiligten klar ersichtlich. Zur Visualisierung wurde ein Prozess-Dashboard definiert (vgl. Abb. 10), das den Arbeitsvorrat jedes einzelnen Prozessschrittes aufzeigt. Im Beispiel ist ersichtlich, dass zum aktuellen Zeitpunkt 601 Corona-infizierte Personen (Indexpersonen) in Quarantäne gesetzt und nun zur weiteren Bearbeitung auf AB_20 stehen. Diese Transparenz unterstützt die Personaleinsatzplanung, weil Bedarfe in den einzelnen Schritten und mögliche Engpässe leicht erkennbar sind.

Zusätzlich zu den Arbeitsvorräten lassen sich auch Analysen der Weiterleitung einzelner Index- und Kontaktpersonen im Sinne eines Process-Mining realisieren. Die automatisch identifizierbaren Prozessabläufe dienen als Input, um Prozessdefinitionen und Arbeitsanweisungen kontinuierlich zu verbessern. So konnte z. B. Ende 2020 ein zeitweiliges Fehlrouting für bestimmte Sonderfälle nach dem Erstanruf von Indexpersonen mittels Process-Mining entdeckt und behoben werden: Zahlreiche Personen waren direkt auf einen späteren Meilenstein gesetzt worden, ohne, dass zuvor relevante Arbeitsschritte durchgeführt worden wären.

### Messbarer Nutzen des Fließbandprinzips

**Abb. 11:**
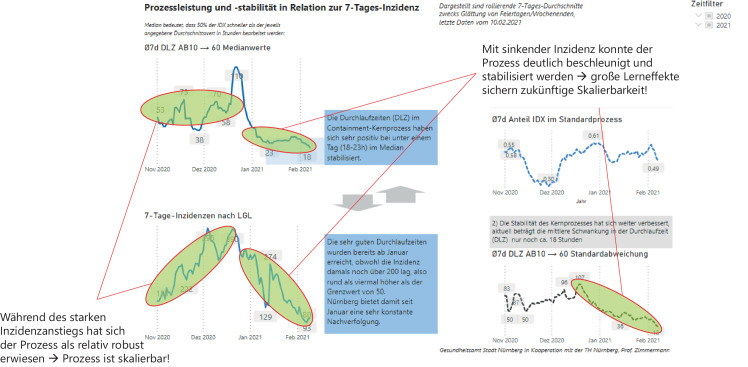
Durchlaufzeitanalyse der Stadt Nürnberg

Auf der Basis der Meilensteinprotokolle erfolgen Durchlaufzeitanalysen, wie in Abb. 11 dargestellt. War die Median-Durchlaufzeit^2^ zu Beginn im November und Dezember noch hoch und schwankend mit etwa zwei bis drei Tagen im 7‑Tages-Mittel^3^, so ging sie seit Januar auf deutlich unter einen Tag zurück. Inhaltlich bedeutet dies, dass mindestens 50 % der Indexpersonen seit Januar in unter einem Tag in Quarantäne gesetzt werden und ihre Kontaktpersonen in dieser Zeit ebenfalls bereits im CCM-System erfasst sind. Der Quarantäneanruf für Kontaktpersonen erfolgt dann in der Regel ebenfalls innerhalb weniger Stunden.

Die Stabilität des Prozesses wird durch die Standardabweichung der Durchlaufzeit bewertet. Diese ist seit Mitte Dezember deutlich auf unter einen Tag gesunken, so dass die Stadt Nürnberg seit Januar ihren Bürger*innen eine verlässliche Reaktionszeit garantieren kann, so sie sich mit Sars-CoV‑2 infizieren. Das ist bemerkenswert, da Nürnberg im Januar zunächst noch über einem Inzidenzwert von 200 lag und dennoch das Containment bereits stabilisiert wurde.

Diese Aussagen können für rund die Hälfte aller Indexpersonen in Nürnberg getroffen werden (vgl. oben rechts in Abb. 11), der andere Teil wird über Sonderteams wie Heimbewohner*innen oder Krankenhauspatient*innen betreut und fließt in diese Analyse nicht ein. Nachdem die Prozessumstellung auf Fließbandprinzip in die Zeit des exponentiellen Anstiegs im November und Dezember 2020 fiel, ist zudem anzumerken, dass sich bereits zu diesem Zeitpunkt die Durchlaufzeiten als relativ robust erwiesen haben (jedoch auf hohem Niveau), obwohl der Prozess noch nicht eingeschwungen war: Ende Oktober war dagegen die Kontaktverfolgung nicht mehr gewährleistet, so dass der neue Prozess von Beginn an ein vollständigeres Containment erlaubte. Die Einschwingphase war letztlich erst nach den Feiertagen Ende 2020 beendet. Dies gibt Anlass zu der Hoffnung, dass der Prozess soweit eingeübt und feinjustiert ist, dass er auch in der dritten Welle gut skalierbar bleibt, sofern das bereits angelernte Personal bei Bedarf reaktiviert werden kann.

## Zweite Delle und dritte Welle – Bundesweite Vernetzung der Gesundheitsämter

### Ungünstiger Zeitpunkt für Systemmigrationen

Mitte April 2021 zum Zeitpunkt der Vollendung dieses Artikels bewegt sich die Inzidenz in Deutschland bereits wieder deutlich über 100. Zugleich hat sich insbesondere eine Mutation des SARS-CoV-2-Virus mit deutlich höherer Ansteckungswirkung und auch höherer Letalität in Deutschland durchgesetzt. Wie aus Abb. 1 ersichtlich, befindet sich Deutschland in einer dritten Welle. Es gab eigentlich fast kein Zeitfenster mit wirklicher Ruhe. Lediglich die Phase von Mitte Januar bis Mitte März 2021 war ein kleines Zeitfenster relativer Ruhe für die Gesundheitsämter und Mitarbeiter*innen, die teilweise seit mehr als einem Jahr auf Hochtouren arbeiten.

Vor diesem Hintergrund ist die geplante Umstellung von gut eingeschwungenen Containment-Prozessen auf neue (IT-)Systeme in der aktuellen Phase als gewagt anzusehen. Allerdings wird von der Politik seit November 2020 genau dies mit dem möglichst raschen Umstieg auf die Software SORMAS gefordert (Bundesregierung 2020). Auch wenn dieses Ziel formal begrüßenswert ist, so verkennen derartige Beschlüsse die Realität komplexer IT-Prozesse und der zugrundeliegenden Softwarelösungen: Die Abbildung existierender Vorgehensweisen auf eine neue Software, das Training hunderter Mitarbeiter*innen auf völlig neue Nutzeroberflächen und Prozesse sowie das Matching und die Migration großer Datenmengen aus den Altsystemen auf die neue Software SORMAS stellen gerade große Kommunen mit eigenen Softwarelösungen vor sehr große Herausforderungen (vgl. z. B. (FAZ 2021)).

So können die Nürnberger Erfahrungen aus der Prozessumstellung seit Oktober 2020 bereits als Indikator dienen, dass eine tiefgreifende Umstellung der Software (anderes Look&Feel) sowie der zugrundeliegenden Prozesse nicht innerhalb weniger Wochen geräuschlos möglich ist, sondern zu deutlichen Verwerfungen in der Effektivität des Containments führen werden (vgl. Durchlaufzeitanalyse in Abb. 11 für die Monate Nov/Dez). Dieses Risiko im Angesicht einer dritten Welle einzugehen und bereits gewonnene Fähigkeiten nicht adäquat einsetzen zu können, scheint aufgrund der zu erwartenden gesellschaftlichen Folgen nicht verantwortbar und sicherlich auch nicht von der Politik intendiert.

### Vernetzungslücke mit SORMAS schließen

**Abb. 12:**
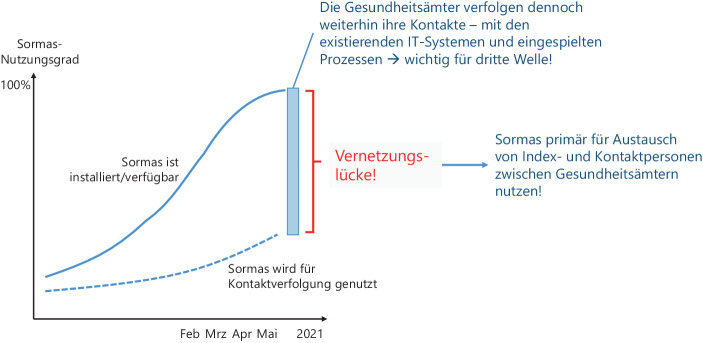
Sormas Installationsrate und produktive Nutzung im Vergleich

Da zumindest die reine Installationsrate für SORMAS in den ersten Wochen des Jahres 2021 deutlich gestiegen ist und in Bayern Ende Februar sogar 100 % erreicht hat (STMGP 2021), die tatsächliche Nutzung der Software für die Kontaktverfolgung jedoch aktuell noch deutlich unterhalb dieser Rate liegt (Mitte März in Bayern laut einer Umfrage bei ca. 30 % (BR24 2021)), ergibt sich eine Lücke, die insbesondere die Vernetzung der Gesundheitsämter untereinander betrifft (vgl. Abb. 12 und Zimmermann (2021)). Um dennoch Nutzen aus der hohen bundesweiten Installationsrate zu ziehen und zugleich kurze Zeitfenster zu nutzen, könnte SORMAS zunächst von Gesundheitsämtern, die nicht wechselfähig sind, nur für den Austausch von Amt zu Amt genutzt werden. Damit könnten Index- und Kontaktpersonen, die formal von anderen Gesundheitsämtern betreut werden müssen, über die einheitlichen Schnittstellen der Version SORMAS X ausgetauscht werden. Gleichzeitig verbleibt die Kontaktverfolgung im eigenen Containment-System.

In Zimmermann (2021) ist exemplarisch erläutert, wie Gesundheitsämter durch relativ überschaubare Ex- und Importe zwischen ihren eigenen Systemen und SORMAS eine Vernetzung erreichen könnten, die aktuell deutschlandweit noch nicht gegeben ist. SORMAS X bietet allen Gesundheitsämtern eine einheitliche Kommunikationsplattform mit gut beschriebenen Schnittstellen sowie Import- und Export-Vorlagen. Jedes Gesundheitsamt muss in diesem Szenario lediglich eine überschaubare Menge an Informationen zu „externen“ Personen (primär Stammdaten, bei Indexpersonen evtl. auch Befunde) mit SORMAS teilen und wird so für alle Gesundheitsämter deutschlandweit einheitlich adressierbar. SORMAS dient in diesem Szenario primär als ein Informations-Hub, das die auszutauschenden Personendaten vereinheitlicht und verteilt.

### Nutzen durch Vernetzung bei Verzicht auf kurzfristige Migrationen zu SORMAS

**Abb. 13:**
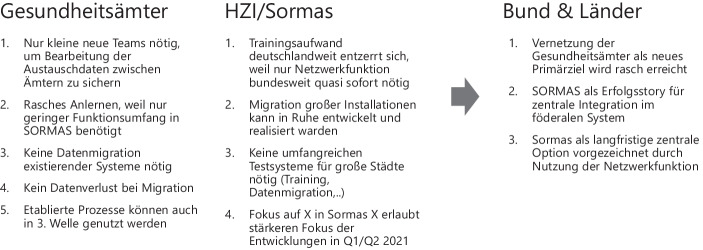
Argumentenbilanz zum Fokus auf Vernetzung der Gesundheitsämter mit SORMAS

Daraus ergeben sich für alle relevanten Anspruchsgruppen (vgl. Abb. 13) kurzfristige Vorteile bei verringerten Aufwänden im Vergleich zu erzwungenen Voll-Migrationen während der dritten Welle. Zugleich wird SORMAS als System deutschlandweit tatsächlich nicht nur installiert, sondern für eine Basisfunktionalität auch kurzfristig genutzt. Dieser Ankerpunkt kann und muss dann in der Folge (ca. ab Sommer 2021, wenn Impfungen hoffentlich weitere Wellen reduzieren) dazu genutzt werden, die Migration der bisherigen lokalen Lösungen auf SORMAS zu realisieren, so dass die vollen Netzwerk- und Synergieeffekte der zentralen Containmentsoftware für den Rest der Pandemie ihren Nutzen entfalten können.

### Software-Lebenszyklus im Zeitraffer

Der vorliegende Artikel kondensiert die Erfahrungen aus einem Jahr Pandemiemanagement im Öffentlichen Gesundheitsdienst. Das Fallbeispiel Nürnberg zeigt, wie schnell dezentrale föderale Einheiten auch digitalisierend reagieren können, wie in der ersten Welle geschehen, als keine zentralen Lösungsoptionen des Bundes oder Landes für die digitale Unterstützung des Containments vorhanden waren. Digitale Systeme alleine bringen jedoch zunächst keinerlei Nutzen, dieser entsteht erst durch eine adäquate organisatorische Einbettung in Arbeitsprozesse. Hier zeigte sich in der zweiten Welle, dass erst durch eine hohe Arbeitsteiligkeit in den Containmentprozessen die Skalierbarkeit (viele Indexfälle) und die Effektivität im Sinne einer verkürzten Durchlaufzeit (rasches Containment und damit Unterbrechung der Infektionsketten) deutlich erhöht werden. Für die nähere Zukunft ist die kritische Frage, wie ein großes System aus Software, Prozessen und Personen auf eine Software neuerer Generation (SORMAS) migriert werden kann, ohne das laufende Containment zu gefährden. Selten ist ein derart kurzer Lebenszyklus von Software, Prozessen und Organisationseinheiten zu beobachten wie im Rahmen dieser Pandemie. In diesem Sinne bietet sich für die Wirtschaftsinformatik neben der praktischen Unterstützung im Pandemiemanagement auch ein fruchtbares Reallabor, das typische Phasen im Lebenszyklus eines Informationssystems von Jahren auf Wochen zusammenkürzt. Welche Kapitel in 2022 rückblickend noch zu schreiben sein werden, ist die offene Frage und der Ansporn für die Weiterentwicklung des Containements in der Corona-Krise.

## Footnotes

1 Beispiele finden sich vielfältig in den Beschlüssen der Ministerpräsidentenkonferenzen und der Länderregierungen.

2 Der Median wird dargestellt, um Verzerrungen durch einzelne Ausreißer in der Bearbeitungszeit zu vermeiden.

3 Der rollierende 7‑Tages-Durchschnitt wird genutzt, um Verzerrungen durch Wochenenden oder Feiertage zu reduzieren, an denen weniger gearbeitet wird.
